# The development of the Compulsive Sexual Behavior Disorder Scale (CSBD-19): An ICD-11 based screening measure across three languages

**DOI:** 10.1556/2006.2020.00034

**Published:** 2020-06-16

**Authors:** Beáta Bőthe, Marc N. Potenza, Mark D. Griffiths, Shane W. Kraus, Verena Klein, Johannes Fuss, Zsolt Demetrovics

**Affiliations:** 1 Institute of Psychology, ELTE Eötvös Loránd University, Budapest, Hungary; 2 Département de Psychologie, Université de Montréal, Montréal, QC, Canada; 3 Yale University School of Medicine, New Haven, CT, USA; 4 Connecticut Council on Problem Gambling, Wethersfield, CT, USA; 5 Connecticut Mental Health Center, New Haven, CT, USA; 6 Psychology Department, International Gaming Research Unit, Nottingham Trent University, Nottingham, UK; 7 Department of Psychology, University of Nevada, Las Vegas, Las Vegas, NV, USA; 8 Institute for Sex Research, Sexual Medicine and Forensic Psychiatry, University Medical Center Hamburg-Eppendorf, Hamburg, Germany

**Keywords:** Compulsive Sexual Behavior Disorder (CSBD), cut-off, hypersexuality, multi-language validation, screening, sex addiction

## Abstract

**Background:**

Compulsive Sexual Behavior Disorder (CSBD) is included in the eleventh edition of *The International Classification of Diseases* (ICD-11) as an impulse-control disorder.

**Aims:**

The aim of the present work was to develop a scale (Compulsive Sexual Behavior Disorder Scale–CSBD-19) that can reliably and validly assess CSBD based on ICD-11 diagnostic guidelines.

**Method:**

Four independent samples of 9,325 individuals completed self-reported measures from three countries (the United States, Hungary, and Germany). The psychometric properties of the CSBD-19 were examined in terms of factor structure, reliability, measurement invariance, and theoretically relevant correlates. A potential threshold was determined to identify individuals with an elevated risk of CSBD.

**Results:**

The five-factor model of the CSBD-19 (i.e., control, salience, relapse, dissatisfaction, and negative consequences) had an excellent fit to the data and demonstrated appropriate associations with the correlates. Measurement invariance suggested that the CSBD-19 functions similarly across languages. Men had higher means than women. A score of 50 points was found as an optimal threshold to identify individuals at high-risk of CSBD.

**Conclusions:**

The CSBD-19 is a short, valid, and reliable measure of potential CSBD based on ICD-11 diagnostic guidelines. Its use in large-scale, cross-cultural studies may promote the identification and understanding of individuals with a high risk of CSBD.

## Introduction

Six years after the exclusion of hypersexual disorder (HD) from the fifth edition of the *Diagnostic and Statistical Manual of Mental Disorders* (DSM-5) (American Psychiatric Association, 2013; Kafka, 2010), Compulsive Sexual Behavior Disorder (CSBD) has been included as a new diagnostic entity in the eleventh edition of *The International Statistical Classification of Diseases* (ICD-11) (World Health Organization, 2019). The inclusion followed extensive theoretical debates about the classification and conceptualization of compulsive sexual behaviors (CSB), and considerable discussion centered on the poor conceptualization of CSB (Fuss et al., 2019). In the ICD-11, CSBD is characterized by a persistent pattern of failure to control intense, repetitive sexual impulses or urges, resulting in repetitive sexual behavior over an extended period (six months or more) that generates marked distress or impairment in personal, family, social, educational, occupational, or other important areas of functioning (Kraus et al., 2018). The diagnostic guidelines of CSBD include several criteria from the previously proposed HD diagnosis, but also important differences, such as a criterion focused on diminished satisfaction and consideration of moral incongruence, reflecting previous criticism regarding the HD diagnosis (Kafka, 2014) (Appendix 1). Although several scales were developed in the past few decades aiming to assess CSB (Bőthe, Kovács, et al., 2019b; Montgomery-Graham, 2017; Stewart & Fedoroff, 2014), no scale exists that assesses CSBD based on ICD-11 guidelines. Thus, the development of a new, valid, and reliable scale (Compulsive Sexual Behavior Disorder Scale–CSBD-19) that assesses the ICD-11 diagnostic guidelines of CSBD (and does not measure prior criteria such as emotion regulation) across different countries (Király et al., 2019) is necessary for both clinical practice and research purposes.

Previous systematic reviews (Karila et al., 2014; Montgomery-Graham, 2017; Womack, Hook, Ramos, Davis, & Penberthy, 2013) reported that more than 30 methods and instruments were used in prior studies to assess CSB with varying reliability and validity. This lack of consistency improved with the publication of the proposed diagnostic criteria for HD (Kafka, 2010), and the assessment of CSB started to converge. As a result, the Hypersexual Behavior Inventory (Reid, Garos, & Carpenter, 2011) was recommended to be used in large-scale survey studies (Bőthe, Kovács et al., 2019b; Karila et al., 2014; Montgomery-Graham, 2017; Womack et al., 2013). HD has been associated with CSB, and many individuals in treatment for HD (>80%) report problems with pornography use (Reid, Carpenter, et al., 2012a). However, with the rejection of HD and the introduction of CSBD in the ICD-11, a valid and reliable measure to assess CSBD is lacking. CSBD may represent a global phenomenon in all genders (Dickenson, Gleason, Coleman, & Miner, 2018) even though cross-cultural (Klein, Jurin, Briken, & Štulhofer, 2015) and gender-based (Bőthe, Bartók et al., 2018a) studies examining CSBD are largely lacking. Although most studies of CSB include predominately male samples and less is known about CSB in women (Klein, Rettenberger, & Briken, 2014), gender-related differences in CSB may be smaller than previously suggested (Dickenson et al., 2018). Thus, it is important to develop a measure to assess CSBD psychometrically equivalently (i.e., demonstrating high levels of measurement invariance) across gender groups and different countries.

### Aims of the present study

The primary aim was to develop a new self-report scale (Compulsive Sexual Behavior Disorder Scale–[CSBD-19]) that can assess CSBD based on ICD-11 diagnostic guidelines/domains (i.e., control, salience, relapse, dissatisfaction, and negative consequences) across cultures and gender groups. We hypothesized that the CSBD-19 would be valid and reliable and demonstrate similar factor structures across three different countries (the United States, Hungary, and Germany) and in both women and men. We further hypothesized that men would show higher scores than women; and across genders, CSBD-19 scores would correlate with measures of hypersexuality and problematic pornography use, and to a lesser extent, with other sexual activities and measures.

## Method

### Participants and procedure

Data were collected via online surveys; completion took approximately 30 minutes. Individuals aged 18 years or older could participate. Regarding *Sample 1*, respondents were invited to participate via an advertisement on a large Hungarian news portal from May to July 2019. Regarding *Sample 2*, a nationally representative probability sample of Hungarians who use the Internet at least once a week was randomly selected from an internet-based panel by a research market company (Solid Data ISA) in May 2019 (for similar methods see Orosz, Bruneau, et al., 2018a). Regarding *Sample 3*, to recruit English-speaking participants, we used Amazon's Mechanical Turk (MTurk)—a reliable data collection platform (Buhrmester, Kwang, & Gosling, 2011)—in August 2019. Between August and September 2019, German-speaking participants were recruited through Internet forums of health care sites and social networks (e.g., Facebook) (Sample 4). Based on prior recommendations for studies conducting factor analysis (VanVoorhis & Morgan, 2007), we aimed to recruit at least 300 participants in each sample to ensure that the analyses would not be underpowered. However, we did not set an upper limit for participation.

#### Sample 1 (Hungarian-speaking community sample)

Of 12,026 individuals who agreed to participate, 55 were excluded for inconsistent response patterns, and 3,976 were excluded for not completing the CSBD-19. Thus, 7,995 individuals (2,815 women, 35.2%) aged between 18 and 76 years (*M*_*age*_ = 36.25 years, *SD*_*age*_ = 12.14) were included. Regarding relationship status, 2,078 reported being single (26.0%), 5,840 reported being in any romantic relationship (i.e., being in a relationship, engaged, or married) (73.1%), and 77 indicated the “other” option (0.9%).

#### Sample 2 (Hungarian-speaking probability sample)

Of 505 individuals who agreed to participate, 32 were excluded for not completing the CSBD-19. Thus, 473 individuals (244 women, 51.6%) aged between 18 and 60 years (*M*_*age*_ = 40.22 years, *SD*_*age*_ = 11.79) were included. Regarding relationship status, 130 reported being single (27.5%), 341 reported being in any romantic relationship (72.1%), and two indicated the “other” option (0.4%).

#### Sample 3 (English-speaking community sample)

Of 538 individuals who agreed to participate, 46 were excluded for inconsistent response patterns, and 15 were excluded for not completing the CSBD-19. Thus, 477 individuals (220 women, 46.1%) aged between 18 and 75 years (*M*_*age*_ = 38.25 years, *SD*_*age*_ = 11.03) were included. Regarding relationship status, 140 reported being single (29.3%), 335 reported being in any romantic relationship (70.2%), and two indicated the “other” option (0.4%).

#### Sample 4 (German-speaking community sample)

Of 541 individuals who agreed to participate, 161 were excluded for not completing the CSBD-19. Thus, 380 individuals (234 women, 61.6%) aged between 18 and 70 years (*M*_*age*_ = 27.81 years, *SD*_*age*_ = 7.73) were included. Regarding relationship status, 99 reported being single (26.0%), 270 reported being in any romantic relationship (71.0%), and 11 indicated the “other” option (3.0%).

### Measures

#### Compulsive Sexual Behavior Disorder Scale (CSBD-19)

The five factors of the CSBD-19 were based on the ICD-11 diagnostic guidelines for CSBD (see Appendix 1): *control* (i.e., failure to control CSB), *salience* (i.e., CSB being the central focus of one's life), *relapse* (i.e., unsuccessful efforts to reduce CSB), *dissatisfaction* (i.e., experiencing less or no satisfaction from sexual behaviors), and *negative consequences* (i.e., CSB generating clinically significant distress or impairment). The negative consequences factor included items related to general and domain-specific adverse consequences. Based on pre-established guidelines (Bőthe, Tóth-Király et al., 2018b; Marsh, Ellis, Parada, Richards, & Heubeck, 2005; Orosz, Tóth-Király, & Bőthe, 2016; Orosz, Tóth-Király et al., 2018b), the authors created and evaluated six items for the control, salience, relapse, and dissatisfaction factors. Given that the negative consequences factor included several domains of negative consequences, six items covering neglect and adverse consequences in general, and three items per each domain covering specific negative consequences were included in the initial item set. When creating the items, the authors also considered potential items from the most frequently used prior scales assessing CSBD-related symptoms (i.e., Hypersexual Behavior Inventory (Reid et al., 2011) and Hypersexual Behavior Consequences Scale (Reid, Garos, & Fong, 2012b)). Before participants indicated their levels of agreement with each item on a four-point scale (1 = "totally disagree", 4 = "totally agree"), they were provided with a definition for "sex" as used in the scale (see Appendix 2). Higher scores on the scale indicate higher levels of CSB. The different language versions are available in Appendix 2.^1^

#### Hypersexual Behavior Inventory–Short Version (HBI-8) (Reid et al., 2011)

The short version of the HBI-8 assesses hypersexuality with eight items. Participants indicated their answers on a five-point scale (1 = “never”; 5 = “very often”). The HBI-8 was registered in three samples and demonstrated excellent reliabilities (α_Sample 1_ = 0.87; α_Sample 3_ = 0.92; α_Sample 4_ = 0.86).

#### Problematic Pornography Consumption Scale-Short Version (PPCS-6) (Bőthe, Tóth-Király, Demetrovics, & Orosz, 2020)

The short version of the PPCS-6 assesses problematic pornography use with six items. Participants indicated their answers on a seven-point scale (1 = “never”; 7 = “all the time”). The PPCS demonstrated excellent reliability in all samples (α_Sample 1_ = 0.86; α_Sample 2_ = 0.88; α_Sample 3_ = 0.87; α_Sample 4_ = 0.82).

#### Sexuality, Masturbation, and Pornography Use-Related Questions (Bőthe, Bartók et al., 2018a)

Respondents indicated the total number of lifetime sexual partners and casual sexual partners (defined as engaging in sexual activities with someone out of a relationship) on 16-point scales (1 = “0”, 16 = “more than 50”). Participants reported their past-year sexual frequencies with their established and casual partners (if they had any), their frequency of masturbation, and their frequency of pornography use on 11-point scales (1 = “never”, 11 = “more than 7 times a week”).

### Statistical analysis

SPSS 25 and Mplus 7.3 were used to conduct statistical analysis. First, the initial item set of the CSBD-19 was examined to select the best items representing each factor based on the combined guidelines of prior work (Marsh et al., 2005; Orosz et al., 2016; Orosz, Tóth-Király et al., 2018b). Confirmatory factor analysis (CFA) was conducted on each sample to cross-validate results. Commonly used goodness-of-fit indices were applied to evaluate models (Hu & Bentler, 1999): Comparative Fit Index (CFI; ≥ .90 acceptable), Tucker–Lewis index (TLI; ≥ .90 acceptable), and Root-Mean-Square Error of Approximation (RMSEA; ≤. 08 acceptable) with its 90% confidence interval. Assumptions of multivariate analyses were examined, and besides normality, all other assumptions were met (see Appendix 3). As compensation for the naturally non-normal distribution of the data, items were treated as categorical indicators, and the mean- and variance-adjusted weighted least-squares estimator (WLSMV) was used (Finney & DiStefano, 2006).

Given that an important point in the assessment of psychological instruments is whether they can be used among individuals from different backgrounds (e.g., different sociodemographic characteristics), it is important to test measurement invariance at high levels (e.g., latent mean invariance) that can ensure the generalizability of the instrument and its constructs (Meredith, 1993; Millsap, 2011; Tóth-Király, Bőthe, Rigó, & Orosz, 2017; Vandenberg & Lance, 2000). For example, if a scale behaves differently in different populations (i.e., high levels of measurement invariance are not achieved), it may lead to measurement biases and invalid comparisons between examined groups. To test measurement invariance between language-based groups (i.e., Hungarian, English, and German) and gender-based groups (i.e., men and women), we conducted multi-group CFAs using each sample (Bőthe, Bartók et al., 2018a; Tóth-Király et al., 2017; Vandenberg & Lance, 2000). Six levels of invariance were tested and compared with increasingly constrained parameters: configural (i.e., factor loadings and threshold were freely estimated), metric (i.e., factor loadings were constrained to be equal), scalar (i.e., factor loadings and threshold were constrained to be equal), residual (i.e., residual variances were constrained to be equal), latent variance-covariance (i.e., factor loadings, thresholds, uniqueness, variances, and covariances were constrained to be equal), and latent mean invariance (i.e., factor loadings, thresholds, uniqueness, variances, covariances, and means were constrained to be equal). Significant decreases in CFI and TLI (ΔCFI ≤ .010; ΔTLI ≤ .010) and significant increases in RMSEA (ΔRMSEA ≤ .015) indicated which level of measurement invariance was achieved (Chen, 2007; Cheung & Rensvold, 2002).

Cronbach's alpha (≥. 70 acceptable) and composite reliability (CR; >.60 acceptable) were calculated to assess the reliability of the CSBD-19. To examine the criterion and convergent validity of the CSBD-19, we assessed associations with theoretically relevant correlates.

To increase the clinical utility of the CSBD-19, we determined a score that could potentially differentiate individuals with and without CSBD. First, we conducted latent profile analysis (LPA) with the robust maximum likelihood estimator on the combined sample to identify a subgroup of individuals who may display symptoms of CSBD (Collins & Lanza, 2010). We used the following indices to determine the number of latent classes based on the factors of CSBD-19: entropy (with higher values indicating higher accuracy), the Akaike Information Criterion (AIC), the bias-corrected Akaike Information Criterion (CAIC), the Bayesian Information Criterion (BIC), and the Sample-Size Adjusted Bayesian Information Criterion (SSABIC) where lower values indicate more parsimonious models. We also used the Lo-Mendell-Rubin Adjusted Likelihood Ratio Test (L-M-R Test) to compare the estimated models. A statistically significant *P*-value (*P* < 0.05) suggests that the model with more classes fits the data better. Second, based on membership in the high-risk group in the LPA, we calculated sensitivity (proportion of true positives belonging to the high-risk group), specificity (proportion of the true negatives belonging to the high-risk group), positive predictive value (proportion of the “true positive” cases: individuals with positive test results who were correctly categorized as being high-risk of CSBD), negative predictive value (proportion of “true negative” cases: individuals with negative test results who were correctly diagnosed as not being high-risk of CSBD), and accuracy values for potential scores on the CSBD-19 (Altman & Bland, 1994a, 1994b; Glaros & Kline, 1988).

### Ethics

The authors assert that all procedures contributing to this work comply with the ethical standards of the relevant national and institutional committees on human experimentation and with the Helsinki Declaration of 1975, as revised in 2008. All procedures involving human subjects/patients were approved by the Research Ethics Committee of the Eötvös Loránd University (2016/286-3) and the Institutional review board of the Centre of Psychosocial Medicine/University Medical Center Hamburg Eppendorf (LPEK-0060). Informed consent was obtained from all participants before enrollment.

## Results

### Item analysis and item reduction

To have a short scale, first, we evaluated each item based on the following criteria (Marsh et al., 2005; Orosz et al., 2016; Orosz, Tóth-Király et al., 2018b): (a) having high corrected item-total correlations, (b) having high standardized factor loadings, (c) having relatively low skewness and kurtoses values, and (d) best covering the breadth of the factor's content (i.e., subjective evaluations from experts in clinical psychology, addiction, sex research, and scale development). Then, we selected those items that represented best the pre-established factors' content and had strong psychometric properties (Appendix 4). As a result, 19 items representing the five pre-established factors of CSBD were retained for further analyses. Three items for the control, salience, relapse, and dissatisfaction factor, and seven items for the negative consequences factor were selected for further analysis.

### The dimensionality, structural validity, and reliability of the CSBD-19

Given the theory-based factors of the CSBD-19 (World Health Organization, 2019) (Appendix 1), CFAs were conducted on the selected items in each sample separately to examine the factor structure of the CSBD-19. The inter-factor correlations in each sample are presented in Appendix 5. The five-factor, first-order model had an excellent fit to the data in each language-based sample (Table 1). The standardized factor loadings and the descriptive statistics of the scale are also presented in Table 2. The CSBD-19 and its factors demonstrated adequate reliability in each sample (Table 2).

**Table 1.** Confirmatory factor analyses (CFA) and tests of invariance on the Compulsive Sexual Behavior Disorder Scale (CSBD-19)

Model	WLSMV χ^2^ (df)	CFI	TLI	RMSEA	90% CI	Comparison	Δχ^2^ (df)	ΔCFI	ΔTLI	ΔRMSEA
5-factor first-order CFA (Sample 1)	7148.851∗(142)	0.944	0.932	0.079	0.077–0.080	—	—	—	—	—
5-factor first-order CFA (Sample 2)	327.290∗(142)	0.983	0.980	0.053	0.045–0.060	—	—	—	—	—
5-factor first-order CFA (Sample 3)	249.477∗(142)	0.994	0.993	0.040	0.032–0.048	—	—	—	—	—
5-factor first-order CFA (Sample 4)	286.037∗(142)	0.967	0.960	0.052	0.043–0.060	—	—	—	—	—
*Language invariance (Sample 1, Sample 2, Sample 3, Sample 4)*										
M1. Configural	7847.926∗(568)	0.948	0.937	0.074	0.073–0.076	–	–	–	–	–	
M2. Metric	7929.214∗(610)	0.948	0.941	0.072	0.070–0.073	M2-M1	204.068∗(42)	0.000	+0.004	−0.002	
M3. Scalar	7146.882∗(709)	0.954	0.956	0.062	0.061–0.064	M3-M2	283.851∗(99)	+0.006	+0.015	−0.010	
M4. Residual	6104.670∗(766)	0.962	0.966	0.055	0.053–0.056	M4-M3	293.405∗(57)	+0.008	+0.010	−0.007	
M5. Latent variance-covariance	3956.990∗(811)	0.978	0.981	0.041	0.040–0.042	M5-M4	372.456∗(45)	+0.016	+0.015	−0.014	
**M6. Latent means**	**3963.853∗(826)**	**0.978**	**0.981**	**0.040**	**0.039–0.042**	**M6-M5**	**111.208∗(15)**	**0.000**	**0.000**	**−0.001**	
*Gender invariance (Merged sample)*										
Baseline men	4806.565∗(142)	0.953	0.943	0.075	0.074–0.077	–	–	–	–	–	
Baseline women	2768.242∗(142)	0.938	0.925	0.073	0.070–0.075	–	–	–`	–	–	
M1. Configural	7406.038∗(284)	0.949	0.938	0.073	0.072–0.075	–	–	–	–	–	
M2. Metric	7603.677∗(298)	0.948	0.940	0.073	0.071–0.074	M2-M1	251.151∗(14)	−0.001	+0.002	0.000	
M3. Scalar	7236.398∗(331)	0.950	0.949	0.067	0.066–0.068	M3-M2	240.306∗(33)	+0.002	+0.009	−0.006	
M4. Residual	6625.373∗(350)	0.955	0.956	0.062	0.061–0.063	M4-M3	217.549∗(19)	+0.005	+0.007	−0.005	
**M5. Latent variance-covariance**	**3111.513∗(365)**	**0.980**	**0.982**	**0.040**	**0.039–0.042**	**M5-M4**	**93.417∗(15)**	**+0.025**	**+0.026**	**−0.022**	
M6. Latent means	5016.435∗(370)	0.967	0.969	0.052	0.051–0.053	M6-M5	839.223∗(5)	−0.013	−0.013	+0.012	

*Note*. WLSMV = weighted least squares mean- and variance-adjusted estimator; χ^2^ = Chi-square; df = degrees of freedom; CFI = comparative fit index; TLI = Tucker-Lewis Index; RMSEA = root-mean-square error of approximation; 90% CI = 90% confidence interval of the RMSEA; ΔCFI = change in CFI value compared to the preceding model; ΔTLI = change in the TLI value compared to the preceding model; ΔRMSEA = change in the RMSEA value compared to the preceding model. Bold letters indicate the final levels of invariance that were achieved. In the language-based comparison, the highest level of measurement invariance (i.e., latent mean invariance) was achieved, indicating that the CSBD-19 functions the same way in each examined language version. In the gender-based comparison, latent variance-covariance was achieved, but latent means invariance was not, indicating important latent mean differences between men and women.∗*P* < 0.001

**Table 2.** Standardized factor loadings, reliability indices, and descriptive statistics of the Compulsive Sexual Behavior Disorder Scale (CSBD-19)

Items	Sample 1 (*N* = 7,995)		Sample 2 (*N* = 473)		Sample 3 (*N* = 477)		Sample 4 (*N* = 380)	
	Factor Loadings							
*Control*								
Even though my sexual behavior was irresponsible or reckless, I found it difficult to stop.	0.771		0.820		0.928		0.597	
I could not control my sexual cravings and desires.	0.803		0.850		0.910		0.694	
My sexual desires controlled me.	0.798		0.898		0.880		0.656	
*Salience*								
Sex has been the most important thing in my life.	0.576		0.547		0.629		0.818	
I would rather have had sex than to have done anything else.	0.829		0.865		0.798		0.871	
When I could have sex, everything else became irrelevant.	0.728		0.732		0.833		0.923	
*Relapse*								
I was able to resist my sexual urges for only a little while before I surrendered to them.	0.845		0.887		0.810		0.815	
Trying to reduce the amount of sex I had almost never worked.	0.855		0.931		0.898		0.915	
I was not successful in reducing the amount of sex I had.	0.901		0.959		0.858		0.953	
*Dissatisfaction*								
I had sex even when I did not enjoy it anymore.	0.881		0.909		0.904		0.908	
Although sex was not as satisfying for me as before, I engaged in it.	0.911		0.945		0.926		0.474	
Although my sex life was not as satisfying as it had been before, I had sex.	0.916		0.930		0.837		0.905	
*Negative Consequences*								
I did not accomplish important tasks because of my sexual behavior.	0.766		0.840		0.819		0.538	
My sexual urges and impulses changed me in a negative way.	0.863		0.931		0.909		0.669	
My sexual activities interfered with my work and/or education.	0.788		0.827		0.863		0.684	
My sexual behaviors had negative impact on my relationships with others.	0.833		0.912		0.888		0.738	
I have been upset because of my sexual behaviors.	0.719		0.847		0.905		0.785	
My sexual activities interfered with my ability to experience healthy sex.	0.744		0.874		0.917		0.854	
I often found myself in an embarrassing situation because of my sexual behavior.	0.747		0.858		0.883		0.794	
	*Reliability Indices*							
	*α*	CR	*α*	CR	*α*	CR	*α*	CR
CSBD-19 total score	0.91	0.97	0.94	0.98	0.94	0.98	0.90	0.97
CSBD-19 control	0.74	0.83	0.81	0.89	0.86	0.93	0.76	0.69
CSBD-19 salience	0.68	0.76	0.65	0.76	0.70	0.80	0.67	0.91
CSBD-19 relapse	0.79	0.90	0.86	0.95	0.78	0.89	0.73	0.92
CSBD-19 dissatisfaction	0.86	0.90	0.89	0.95	0.85	0.92	0.87	0.82
CSBD-19 negative consequences	0.83	0.92	0.90	0.96	0.92	0.96	0.80	0.89
	*Mean (SD)*							
CSBD-19 total score	1.52 (0.50)		1.44 (0.51)		1.49 (0.54)		1.45 (0.43)	
CSBD-19 control	1.59 (0.70)		1.42 (0.66)		1.40 (0.67)		1.35 (0.56)	
CSBD-19 salience	1.80 (0.71)		1.64 (0.66)		1.58 (0.64)		1.71 (0.63)	
CSBD-19 relapse	1.46 (0.65)		1.35 (0.61)		1.51 (0.68)		1.40 (0.56)	
CSBD-19 dissatisfaction	1.44 (0.66)		1.48 (0.73)		1.62 (0.79)		1.45 (0.67)	
CSBD-19 negative consequences	1.48 (0.48)		1.30 (0.52)		1.35 (0.60)		1.32 (0.44)	

*Note*. All factor loadings are standardized. Loadings are statistically significant at *P* < 0.001. CSBD-19 = Compulsive Sexual Behavior Disorder Scale; CI = confidence interval; SD = standard deviation; CR = composite reliability.

To lend further support for the validity of the CSBD-19 and to ensure that language-based comparisons are meaningful, we examined the invariance of the factor structure of the CSBD-19 across the four samples. Baseline models were estimated for each group and, then, parameters were gradually constrained. The fit indices suggested that the highest level of invariance (latent mean invariance) was achieved, indicating that the CSBD-19 appears to function the same way in each language version (Table 1). Next, we conducted measurement invariance testing to examine the factor structure of the CSBD-19 across genders (men vs. women) on a combined sample, including samples 1–4. Fit indices suggested that latent variance-covariance invariance was achieved, but latent mean invariance was not, suggesting the presence of latent mean differences between men and women (Table 1). Using the variance-covariance model, latent mean differences between men and women are expressed in SD units and are accompanied by tests of statistical significance. When men's latent means were constrained to zero for the purpose of model identification, women's latent means proved to be substantially lower on all factors (Control: −0.47 SD, *P* < 0.001; Salience: −0.59 SD, *P* < 0.001; Relapse: −0.65 SD, *P* < 0.001; Negative Consequences: −0.31 SD, *P* < 0.001) except for the Dissatisfaction factor (0.01 SD, *P* = 0.612).

### The associations of the CSBD-19 with theoretically relevant correlates

Regarding convergent and criterion validity, the CSBD-19 scores had strong, positive associations with HBI-8 and PPCS-6 scores and weak-to-moderate, positive associations with frequencies of pornography use, masturbation, and having sex with casual partners in each sample. CSBD-19 scores had weak, positive associations with the numbers of sexual partners and casual sexual partners in one's lifetime in each sample. However, CSBD-19 scores were unrelated or weakly and negatively related to the frequency of past-year sexual activity with one's partner (Table 3).

**Table 3.** Associations between the Compulsive Sexual Behavior Disorder Scale (CSBD-19) and theoretically relevant correlates

	Sample 1 (*N* = 7,995, *N*^c^ = 5,840, *N*^d^ = 2,949)	Sample 2 (*N* = 473, *N*^c^ = 341)	Sample 3 (*N* = 477, *N*^c^ = 335, *N*^d^ = 96)	Sample 4 (*N* = 380, *N*^c^ = 270, *N*^d^ = 134)
Hypersexual Behavior Inventory-Short Version (HBI-8)	0.75*	–	0.81*	0.79*
Problematic Pornography Consumption Scale-Short Version (PPCS-6)	0.55*	0.53*	0.69*	0.60*
Number of sexual partners^a^	0.17*	0.18*	0.12*	0.09
Number of casual sexual partners^a^	0.21*	0.22*	0.22*	0.17*
Past-year frequency of having sex with the partner^b^	−0.04*	0.03	−0.16*	−0.01
Past-year frequency of having sex with casual partners^b,e^	0.12*	0.19*	−0.03	0.02
Past-year frequency of masturbation^b^	0.27*	–	0.20*	0.32*
Past-year frequency of pornography viewing^b^	0.29*	0.29*	0.23*	0.40*

*Note*. * *P* < 0.01.

^a^1 = 0 partner; 2 = 1 partner; 3 = 2 partners; 4 = 3 partners; 5 = 4 partners; 6 = 5 partners; 7 = 6 partners; 8 = 7 partners; 9 = 8 partners; 10 = 9 partners; 11 = 10 partners; 12 = 10 partners; 12 = 11–20 partners, 13 = 21–30 partners; 14 = 31–40 partners; 15 = 41–50 partners; 16 = more than 50 partners.

^b^1 = never; 2 = once in the last year; 3 = 1–6 times in the last year; 4 = 7–11 times in the last year; 5 = monthly; 6 = two or three times a month; 7 = weekly; 8 = two or three times a week; 9 = four or five times a week; 10 = six or seven times a week; 11 = more than seven times a week.

^c^Number of partnered respondents.

^d^Number of respondents who had casual sexual partners.

^e^In the case of Sample 2, everyone answered to this question, not only those participants who had ever had casual sexual partners.

### Determination of a potential threshold score for the CSBD-19

First, latent profile analysis was conducted on the five factors of the CSBD-19 in the combined sample. The AIC, BIC, and SSABIC values continuously decreased as more latent classes were added, and all solutions had high levels of accuracy (based on entropy). The L-M-R Test suggested that the six-class solution should be favored in contrast to the seven-class solution; thus, we used these six classes in further analysis (see Appendix 6). The *fourth class* (high-risk class; 260 participants, 2.8%) represented individuals with being at high-risk of CSBD (Appendix 7). The characteristics of the identified classes are presented in Table 4. The high-risk class demonstrated significantly higher scores on the CSBD-19 (with having the highest score differences on the negative consequences factor), HBI-8, and PPCS-6 than the other classes. The high-risk class had the highest number of lifetime sexual partners and casual sexual partners and the highest frequency of past-year masturbation and pornography use.

**Table 4.** Comparison of the Compulsive Sexual Behavior Disorder Scale (CSBD-19) score-based latent classes on theoretically relevant key constructs (*N* = 9,325)

	1. Dissatisfied low-risk class (7.2%)M (SD)	2. Low-risk class (60.3%)M (SD)	3. Average-risk class (17.5%)M (SD)	4. High-risk class (2.8%)M (SD)	5. At-risk class (7.8%)M (SD)	6. Satisfied at-risk class (4.5%)M (SD)	ANOVA	
							*F*	*η* ^2^
CSBD-19	29.84 (4.06)^2,3,4,5,6^	22.36 (2.83)^1,3,4,5,6^	32.29 (3.25)^1,2,4,5,6^	56.74 (5.33)^1,2,3,5,6^	42.47 (4.09)^1,2,3,4,6^	43.35 (4.63)^1,2,3,4,5^	12400.94*	0.87
HBI-8	1.70 (0.55)^2,3,4,5,6^	1.39 (0.42)^1,3,4,5,6^	2.03 (0.58)^1,2,4,5,6^	3.52 (0.75)^1,2,3,5,6^	2.51 (0.71)^1,2,3,4,6^	2.81 (0.67)^1,2,3,4,5^	1588.33*	0.51
PPCS-6	2.12 (1.37)^2,3,4,5,6^	1.72 (1.00)^1,3,4,5,6^	2.50 (1.41)^1,2,4,5,6^	4.56 (2.56)^1,2,3,5,6^	3.28 (1.88)^1,2,3,4^	3.43 (1.90)^1,2,3,4^	389.63*	0.22
Number of sexual partners^a^	8.47 (4.32)^3,4,5,6^	8.25 (4.37)^3,4,5,6^	9.50 (4.35)^1,2^	10.17 (4.63)^1,2^	9.75 (4.48)^1,2^	9.84 (4.52)^1,2^	42.97*	0.02
Number of casual sexual partners^a^	5.70 (4.65)^3,4,5,6^	5.34 (4.54)^3,4,5,6^	6.95 (4.85)^1,2,4^	8.32 (5.25)^1,2,3^	7.41 (5.02)^1,2^	7.58 (5.07)^1,2^	71.62*	0.04
Past-year frequency of having sex with the partner^b^	6.59 (1.79)	6.85 (1.86)^5^	6.86 (2.10)^5^	6.39 (2.46)	6.43 (2.03)^2,3^	6.57 (2.19)	7.31*	0.01
Past-year frequency of having sex with casual partners^b^	3.55 (2.12)^3,4,5,6^	3.67 (2.23)^3,4,5,6^	4.10 (2.13)^1,2^	4.51 (2.23)^1,2^	4.15 (2.17)^1,2^	4.56 (2.20)^1,2^	14.79*	0.02
Past-year frequency of masturbation^b^	6.55 (2.50)^3,4,5,6^	6.54 (2.39)^3,4,5,6^	7.59 (2.23)^1,2,4,5,6^	8.47 (2.29)^1,2,3,5^	7.91 (2.21)^1,2,3,4^	8.07 (2.16)^1,2,3^	120.67*	0.06
Past-year frequency of pornography viewing^b^	5.46 (2.82)^3,4,5,6^	5.67 (2.87)^3,4,5,6^	7.09 (2.63)^1,2,4,6^	7.97 (2.72)^1,2,3^	7.42 (2.64)^1,2^	7.73 (2.55)^1,2,3^	137.01*	0.08

*Note*. M = mean; SD = standard deviation; CSBD-19 = Compulsive Sexual Behavior Disorder Scale; HBI-8 = Hypersexual Behavior Inventory-Short Version; PPCS-6 = Problematic Pornography Consumption Scale-Short Version.

*η*^2^ = Eta-squared. Superscript numbers (1, 2, 3, 4, 5, 6) indicate significant (*P* < 0.05) difference between the given class and the indexed group within the same variable. * *P* < 0.001

^a^1 = 0 partner; 2 = 1 partner; 3 = 2 partners; 4 = 3 partners; 5 = 4 partners; 6 = 5 partners; 7 = 6 partners; 8 = 7 partners; 9 = 8 partners; 10 = 9 partners; 11 = 10 partners; 12 = 10 partners; 12 = 11–20 partners, 13 = 21–30 partners; 14 = 31–40 partners; 15 = 41–50 partners; 16 = more than 50 partners.

^b^1 = never; 2 = once in the last year; 3 = 1–6 times in the last year; 4 = 7–11 times in the last year; 5 = monthly; 6 = two or three times a month; 7 = weekly; 8 = two or three times a week; 9 = four or five times a week; 10 = six or seven times a week; 11 = more than seven times a week.

Based on membership in the high-risk class as a “gold standard”, the sensitivity, specificity, positive predictive value (PPV), negative predictive value (NPV), and accuracy of potential threshold scores were calculated for the CSBD-19 (Appendix 8). A score of 50 points was suggested as an optimal cut-off to be classified as being at high-risk of CSBD. For this threshold, the sensitivity was 98.5%, the specificity was 99.1%, the PPV was 76.4%, the NPV was 100%, and the accuracy was 99.1%. These results practically mean that 0.9% of low-risk individuals were misidentified as high-risk individuals, while 1.5% of the “true” high-risk individuals were not recognized by the CSBD-19. Approximately one-quarter of the individuals with a positive test result (having ≥ 50 scores on the CSBD-19) was mistakenly identified as high-risk individuals; however, almost everyone with a negative result (having scores <50) were identified correctly as low-risk individuals. Using the established threshold, 4.2% of men and 2.0% of women in Sample 1; 5.2% of men and 3.3% of women in Sample 2; 7.0% of men and 5.5% of women in Sample 3; and 5.6% of men and 0% of women were classified as having high-risk for CSBD.

## Discussion

A measure for assessing ICD-11-defined CSBD (World Health Organization, 2019) is necessary to address current gaps in the treatment and research. We developed the CSBD-19 and tested its psychometric properties across three languages in four samples, demonstrating robust psychometric properties in terms of factor structure, reliability, measurement invariance, and associations with theoretically relevant constructs. A threshold was determined that can identify individuals at high-risk of CSBD. Initial findings suggest the CSBD-19 may have clinical utility, although further research is needed to test and refine the CSBD-19 with clinical and nonclinical samples.

The construct validity and reliability of CSBD-19 were cross-validated in three languages in four independent samples from the United States, Hungary, and Germany. Not only was the construct validity of CSBD-19 supported, but also its convergent validity was also established by its positive, strong association with the HBI-8 (Reid et al., 2011). In line with previous findings, CSBD-19 scores demonstrated positive, strong associations with measures of problematic pornography use (Bőthe, Koós, Tóth-Király, Orosz, & Demetrovics, 2019a; Bőthe, Tóth-Király et al., 2019c), and positive, weak-to-moderate associations with the past-year frequency of pornography use, past-year frequency of masturbation, and the number of lifetime sexual and casual sexual partners (Bőthe, Kovács et al., 2019b). The frequency of past-year sexual activities with one's partner was unrelated to the CSBD-19 scores, in line with prior findings from large-scale studies (Štulhofer, Jurin, & Briken, 2016).

High levels of measurement invariance were demonstrated across language-based and gender-based groups. In the case of language-based groups, the highest level of invariance was achieved, suggesting that the CSBD-19 may be used reliably in future cross-cultural studies assessing CSBD and the differences in CSBD scores may be attributed to actual differences between the language-based samples, and not to methodological shortcomings (Király et al., 2019).

Prevalence estimates for being at high-risk for CSBD varied between 0–5.5% for women and 4.2–7% for men in the present study. The observed variation in prevalence rates across countries may be in part explained by the different recruitment methods used (i.e., news portal, research panel, and social media). However, the results support the notion that gender-related differences in CSBD may be smaller than existing data may suggest (Dickenson et al., 2018; Erez, Pilver, & Potenza, 2014). Previous research shows that gender norms may influence sexual desire in women (Rubin et al., 2019), and suggests a possible role for moral incongruence in self-reported problems with CSB (Grubbs, Perry, Wilt, & Reid, 2019). Different prevalence estimates, especially among women, may be related to differences in gender and sexual norms, moral values, and religiosity among the three countries. Although this explanation is rather speculative, future research should examine this possibility. The scale, nevertheless, demonstrated high levels of reliability and validity among both men and women and may be used in men and women, although further testing with women is recommended.

Based on the results of the LPA, six groups were identified and could be reliably distinguished based on their CSBD characteristics. Approximately 85% of the participants belonged to the low- and average-risk classes. Individuals in these classes also reported lower levels of lifetime and past-year sexual activities (e.g., number of lifetime sexual partners or past-year pornography use frequency) than participants in the at-risk and high-risk classes. A minority (7.8%) of participants was included in the at-risk class; these participants demonstrated slightly elevated levels of CSBD compared to the average-risk class. Two higher-risk groups were identified. The first (i.e., satisfied at-risk class) included 4.5% of participants, and they reported elevated levels on all factors of the CSBD-19 except for the dissatisfaction criterion. The findings suggest that these individuals may experience uncontrollable sexual activities, but they are not dissatisfied with their sexual activities, and they do not experience as many negative consequences as people in the high-risk class. These individuals may have higher levels of sexual desire that may result in some similar characteristics as CSBD, but without some important indicators of CSBD (Štulhofer et al., 2016). Lastly, a high-risk group of CSBD (2.8%) was identified who also demonstrated the highest levels of problematic pornography use and other sexual activities. The percentage of high-risk individuals is in line with prior estimates that suggest that CSBD could be experienced by 1–10% of the general adult population (Montgomery-Graham, 2017).

Finally, the sensitivity and specificity analyses and the positive and negative predictive values suggest an optimal threshold score of 50 points (out of 76 points) that may identify individuals at high-risk of CSBD. Despite the high accuracy of the recommended cut-off score, it should be noted that only community samples (i.e., not clinical samples) were examined in the present study. Moreover, self-report scales (such as the CSBD-19) should only be used as a first step (screening) of the diagnostic process followed by clinical interviews (Bőthe, Kovács et al., 2019b). Future studies should further validate this threshold in treatment-seeking clinical samples to extend the present findings and provide evidence for the clinical validity and utility of the CSBD-19.

To summarize, the CSBD-19 was developed by following rigorous guidelines, yielded strong psychometric properties in three languages in four large samples, and showed differentiated results in the case of individuals with and without high-risk of CSBD. Despite its strengths, the present study had some limitations that should be noted. The study used cross-sectional, self-reported data; thus, the results may be prone to biases (e.g., social desirability). Also, the study was conducted using only community samples; therefore, the clinical validity and utility of the CSBD-19 require further investigation. Future studies are needed to examine the construct validity of CSBD-19 conducting within-network and between-network studies on different populations, such as in clinical settings, or in different cultures, considering the potential role of moral incongruence in perceived CSBD (Grubbs et al., 2019). Although the CSBD-19 was developed in an international setting and its psychometric properties were tested in Europe and the US as well, the present study is only the first step in a thorough examination of the CSBD-19. Future studies are needed to examine the reliability and the validity of the CSBD-19 in other countries and cultures (e.g., Eastern cultures) (Chen & Jiang, 2020).

### Conclusions and implications

The CSBD-19 is a short, valid, and reliable measure of CSBD based on ICD-11 diagnostic guidelines (World Health Organization, 2019). It can be included in large-scale, cross-cultural, multi-language studies, and can reliably distinguish between individuals at elevated and lower risk of CSBD. The use of the CSBD-19 should help to identify and study individuals with CSBD. Thus, the incomparability of findings (Karila et al., 2014; Montgomery-Graham, 2017; Womack et al., 2013)—a major problem in research addressing compulsive, impulsive, and addictive sexual behaviors—may be eliminated, and cross-cultural research on CSBD may be facilitated.

## Acknowledgments and funding sources

The research was supported by the Hungarian National Research, Development, and Innovation Office (Grant numbers: KKP126835, NKFIH-1157-8/2019-DT). BB was supported by a postdoctoral fellowship award by the SCOUP Team—Sexuality and Couples—Fonds de recherche du Québec, Société et Culture. Dr. Potenza receives support from the Connecticut Department of Mental Health and Addiction Services, the Connecticut Council on Problem Gambling, the Connecticut Mental Health Center, and the National Center for Responsible Gaming. The funding agencies did not have input into the content of the manuscript, and the views described in the manuscript reflect those of the authors and not necessarily those of the funding agencies.

## Author's contributions

*Category 1:* a) conception and design: BB; MNP; MDG; SWK; VK; JF; ZD b) analysis of data: BB c) interpretation of data: BB; MNP; MDG; SWK; VK; JF; ZD. *Category 2:* a) drafting the article: BB b) revising it critically for important intellectual content: BB; MNP; MDG; SWK; VK; JF; ZD*. Category 3:* a) final approval of the version to be published: BB; MNP; MDG; SWK; VK; JF; ZD.

## Conflict of interests

The authors declare no conflict of interest with respect to the content of this manuscript.

## Supplementary Material

## Footnotes

1 The scale was developed in English and Hungarian simultaneously. Then, the English version was translated to German, and back-translated to English by a native speaker who was unaware of the original version. The developers of the original versions (English and Hungarian) checked the back-translation, compared it to the original version, and approved it.
