# Supplementary material for: The development of the Compulsive Sexual Behavior Disorder Scale (CSBD-19): An ICD-11 based screening measure across three languages
Source: J Behav Addict. 2020 Jun 16;9(2):247–58. doi: 10.1556/2006.2020.00034 (PMC8939427; doi:10.1556/2006.2020.00034)
Supplement: Supplementary file 1 [file jba-9-247-s001.docx]

SUPPLEMENTAL MATERIAL FOR:

**The Development of the Compulsive Sexual Behavior Disorder Scale (CSBD-19): An ICD-11 Based Screening Measure Across Three Languages**

Appendix 1. Table 1. Comparison of diagnosis criteria for hypersexual disorder (HD) and compulsive sexual behavior disorder (CSBD)

| **Diagnostic Criteria** | **Hypersexual Disorder**  **(Kafka, 2010)** | **Compulsive Sexual Behavior Disorder (ICD-11, 2019)** |
| --- | --- | --- |
| Control | - over a period of at least six months, recurrent and intense sexual fantasies, sexual urges, or sexual behaviors | - persistent pattern of failure to control intense, repetitive sexual impulses or urges resulting in repetitive sexual behavior |
| Salience | — | - repetitive sexual activities becoming a central focus of the person’s life |
| Relapse | - repetitive but unsuccessful efforts to control or signiﬁcantly reduce these sexual fantasies, urges or behaviors | - numerous unsuccessful efforts to significantly reduce repetitive sexual behavior |
| Dissatisfaction | — | - continued repetitive sexual behavior despite deriving little or no satisfaction from sexual behavior |
| Negative consequences | - there is clinically signiﬁcant personal distress or impairment in social, occupational or other important areas of functioning associated with the frequency and intensity of these sexual fantasies, urges or behaviors - repetitively engaging in sexual behaviors while disregarding the risk for physical or emotional harm to self or others - time consumed by sexual fantasies, urges or behaviors repetitively interferes with other important (non-sexual) goals, activities, and obligations | - continued repetitive sexual behavior despite adverse consequences - neglecting health and personal care or other interests, activities, and responsibilities - generates marked distress or significant impairment in personal, family, social, educational, occupational, or other important areas of functioning |
| In response to negative emotions | - repetitively engaging in sexual fantasies, urges or behaviors in response to dysphoric mood states (e.g., anxiety, depression, boredom, irritability) | — |
| In response to stress | - repetitively engaging in sexual fantasies, urges or behaviors in response to stressful life events | — |
| Not due to other problems | - these sexual fantasies, urges or behaviors are not due to the direct physiological effect of an exogenous substance (e.g., a drug of abuse or a medication) | - exclusions: paraphilic disorders, medical conditions (e.g., dementia), substance use, due to medication (see (Kraus et al., 2018)) - distress that is entirely related to moral judgments and disapproval about sexual impulses, urges, or behaviors is not sufficient to meet this requirement |
| Subcategories | - masturbation, pornography, sexual behavior with consenting adults, cybersex, telephone sex, strip clubs | — |

*Note.* The diagnostic criteria and their literal descriptions are based on the proposed but rejected diagnosis of hypersexual disorder (HD; Kafka, 2010) and the accepted diagnostic criteria of compulsive sexual behavior disorder (CSBD; World Health Organization, 2019). The table is based on a prior comparison of HD and CSBD

Appendix 2. Compulsive Sexual Behavior Disorder Scale (CSBD-19) Versions

**CSBD-19 – Hungarian Version**

Az alábbi állítások különböző gondolatokat, érzéseket és viselkedéseket írnak le a szexszel kapcsolatban. Gondolj vissza az **előző hat hónapra** és jelöld egy négyfokú skálán, hogy mennyire jellemzőek rád az alábbi álllítások! Nincsenek jó vagy rossz válaszok!

*A kérdőív szexnek tekint minden olyan cselekvést vagy viselkedést, amely stimulál vagy felizgat valakit és célja a szexuális gyönyör vagy orgazmus elérése (pl. önkielégítés, pornográfia nézése, partnerrel való szexuális együttlét bármely formája, közösülés, orális szex, anális szex stb.). Ne feledd tehát, hogy szexuális viselkedés egyaránt létrejöhet egyedül és partnerrel!*

| *1 –*  *egyáltalán nem értek egyet* | *2 –*  *inkább nem értek egyet* | *3 –*  *inkább egyetértek* | *4 –*  *teljes mértékben egyetértek* |
| --- | --- | --- | --- |

|  | 1 | 2 | 3 | 4 |
| --- | --- | --- | --- | --- |
| 1. Bár szexuális viselkedésem felelőtlen és meggondolatlan volt, nehezemre esett ellenállni. | O | O | O | O |
| 1. A szex volt a legfontosabb dolog az életemben. | O | O | O | O |
| 1. Csak rövid ideig tudtam ellenállni a szexuális késztetéseimnek, mielőtt feladtam. | O | O | O | O |
| 1. Még akkor is szexeltem, amikor már egyáltalán nem élveztem azt. | O | O | O | O |
| 1. A szexuális késztetéseim és ösztöneim rossz irányba változtattak meg engem. | O | O | O | O |
| 1. Nem tudtam szabályozni a szexuális sóvárgásomat és vágyaimat. | O | O | O | O |
| 1. Inkább szexeltem, minthogy bármi mást csináltam volna. | O | O | O | O |
| 1. Alig működött az, ha megpróbáltam csökkenteni, hogy mennyit szexelek. | O | O | O | O |
| 1. Habár annyira már nem volt kielégítő a szex, mint korábban, mégis csináltam. | O | O | O | O |
| 1. A szexuális viselkedésem miatt nem teljesítettem fontos feladatokat. | O | O | O | O |
| 1. A szexuális vágyaim irányítottak engem. | O | O | O | O |
| 1. Amikor szexelhettem, minden más jelentéktelenné vált. | O | O | O | O |
| 1. Nem tudtam sikeresen csökkenteni, hogy mennyit szexelek. | O | O | O | O |
| 1. Annak ellenére is szexeltem, hogy a szexuális életem nem volt már olyan kielégítő, mint korábban. | O | O | O | O |
| 1. A szexuális viselkedésem akadályozott a munkában és/vagy a tanulmányaimban. | O | O | O | O |
| 1. A szexuális viselkedésem negatívan hatott az emberekkel való kapcsolatomra. | O | O | O | O |
| 1. Nagyon szomorú voltam a szexuális viselkedésem miatt. | O | O | O | O |
| 1. Előfordult már, hogy a szexuális viselkedésem korlátozott az egészséges szexuális élmény átélésében. | O | O | O | O |
| 1. Gyakran kellemetlen helyzetben találtam magam a szexuális viselkedésem miatt. | O | O | O | O |

**Kiértékelés:** tételek pontszámát össze kell adni. 50 pont vagy annál magasabb pontszám a kompulzív szexuális viselkedés magas kockázatát jelzi.

**A kérdőív faktorai:**

*Kontroll:* 1., 6., 11.

*Szaliencia:* 2., 7., 12.

*Visszaesés:* 3., 8., 13.

*Elégedetlenség:* 4., 9., 14.

*Negatív következmények:* 5., 10., 15., 16., 17., 18., 19.

**CSBD-19 – English Version**

Below are a number of statements that describe various thoughts, feelings, and behaviors about sex. Please, think back to the **past six months** and indicate on the following 4-point scale to what extent the statements apply to you. There are no right or wrong answers.

*For the purpose of this questionnaire, sex is defined as any activity or behavior that stimulates or arouses a person with the intent to produce an orgasm or sexual pleasure (e.g., self-masturbation or solosex, using pornography, intercourse with a partner, oral sex, anal sex, etc.). Sexual behaviors may or may not involve a partner.*

| *1 –*  *totally disagree* | *2 –*  *somewhat disagree* | *3 –*  *somewhat agree* | *4 –*  *totally agree* |
| --- | --- | --- | --- |

|  | 1 | 2 | 3 | 4 |
| --- | --- | --- | --- | --- |
| 1. Even though my sexual behavior was irresponsible or reckless, I found it difﬁcult to stop. | O | O | O | O |
| 1. Sex has been the most important thing in my life. | O | O | O | O |
| 1. I was able to resist my sexual urges for only a little while before I surrendered to them. | O | O | O | O |
| 1. I had sex even when I did not enjoy it anymore. | O | O | O | O |
| 1. My sexual urges and impulses changed me in a negative way. | O | O | O | O |
| 1. I could not control my sexual cravings and desires. | O | O | O | O |
| 1. I would rather have had sex than to have done anything else. | O | O | O | O |
| 1. Trying to reduce the amount of sex I had almost never worked. | O | O | O | O |
| 1. Although sex was not as satisfying for me as before, I engaged in it. | O | O | O | O |
| 1. I did not accomplish important tasks because of my sexual behavior. | O | O | O | O |
| 1. My sexual desires controlled me. | O | O | O | O |
| 1. When I could have sex, everything else became irrelevant. | O | O | O | O |
| 1. I was not successful in reducing the amount of sex I had. | O | O | O | O |
| 1. Although my sex life was not as satisfying as it had been before, I had sex. | O | O | O | O |
| 1. My sexual activities interfered with my work and/or education. | O | O | O | O |
| 1. My sexual behaviors had negative impact on my relationships with others. | O | O | O | O |
| 1. I have been upset because of my sexual behaviors. | O | O | O | O |
| 1. My sexual activities interfered with my ability to experience healthy sex. | O | O | O | O |
| 1. I often found myself in an embarrassing situation because of my sexual behavior. | O | O | O | O |

**Scoring:** Add the scores of the items. 50 points or more indicate high risk of compulsive sexual behavior disorder.

**Factors of the scale:**

*Control:* 1., 6., 11.

*Salience:* 2., 7., 12.

*Relapse:* 3., 8., 13.

*Dissatisfaction:* 4., 9., 14.

*Negative consequences:* 5., 10., 15., 16., 17., 18., 19.

**CSBD-19 – German Version**

Unten stehen einige Aussagen, die verschiedene Gedanken und Gefühle über Sex sowie sexuelle Verhaltensweisen beschreiben. Bitte denken Sie an die **letzten 6 Monate** zurück und geben Sie auf der vierstufigen Skala an, in welchem Maß die Aussagen auf Sie zutreffen. Es gibt keine richtigen oder falschen Antworten.

*Für diesen Fragebogen ist Sex definiert als jede Aktivität oder jedes Verhalten, das eine Person stimuliert oder erregt, mit dem Ziel, einen Orgasmus oder sexuelles Vergnügen hervorzurufen (z.B. Selbstbefriedigung oder Solo-Sex, Konsum von Pornografie, Geschlechtsverkehr mit Partnerin oder Partner, Oralverkehr, Analverkehr etc…). Sexuelle Verhaltensweisen können mit oder ohne Partnerin/Partner stattfinden.*

| *1 –*  *Stimme überhaupt nicht zu* | *2 –*  *Stimme eher nicht zu* | *3 –*  *Stimme eher zu* | *4 –*  *Stimme voll zu* |
| --- | --- | --- | --- |

|  | 1 | 2 | 3 | 4 |
| --- | --- | --- | --- | --- |
| 1. Obwohl mein Sexualverhalten unverantwortlich oder leichtsinnig war, fand ich es schwierig, aufzuhören. | O | O | O | O |
| 1. Sex war die wichtigste Sache in meinem Leben. | O | O | O | O |
| 1. Ich konnte meinem sexuellen Verlangen nur eine Weile widerstehen, bevor ich ihm nachgab. | O | O | O | O |
| 1. Ich hatte Sex, selbst wenn ich es nicht mehr genießen konnte. | O | O | O | O |
| 1. Mein sexuelles Verlangen und sexuellen Impulse haben mich auf eine negative Art verändert. | O | O | O | O |
| 1. Ich konnte mein sexuelles Verlangen und Begehren nicht kontrollieren. | O | O | O | O |
| 1. Ich hätte lieber Sex gehabt als irgendetwas anderes zu tun. | O | O | O | O |
| 1. Mein Versuch, die Menge an Sex, die ich hatte, zu verringern hat fast nie funktioniert. | O | O | O | O |
| 1. Obwohl Sex für mich nicht mehr so befriedigend war wie früher, ließ ich mich darauf ein. | O | O | O | O |
| 1. Ich habe wichtige Aufgaben wegen meines Sexualverhaltens nicht erledigt. | O | O | O | O |
| 1. Meine sexuellen Wünsche und Impulse kontrollierten mich. | O | O | O | O |
| 1. Wenn ich Sex haben konnte, wurde alles andere irrelevant. | O | O | O | O |
| 1. Ich war nicht erfolgreich dabei, die Menge an Sex, die ich hatte, zu verringern. | O | O | O | O |
| 1. Obwohl mein Sexualleben nicht mehr so befriedigend war wie vorher, hatte ich Sex. | O | O | O | O |
| 1. Meine sexuellen Aktivitäten störten meine Arbeit und/oder mein Studium (bzw. Schule/Ausbildung). | O | O | O | O |
| 1. Mein Sexualverhalten hatte negativen Einfluss auf meine Beziehungen zu anderen. | O | O | O | O |
| 1. Ich war wegen meines Sexualverhaltens aufgebracht. | O | O | O | O |
| 1. Meine sexuellen Aktivitäten störten meine Fähigkeit, gesunden Sex zu haben. | O | O | O | O |
| 1. Ich fand mich wegen meines Sexualverhaltens oft in einer peinlichen Situation wieder. | O | O | O | O |

**Auswertung:** Addieren Sie die Summe der Items. Ein Wert von 50 oder höher bedeutet ein hohes Risiko für das Vorliegen einer CSBD.

**Faktoren der Skala:**

*Kontrolle:* 1., 6., 11.

*Vereinnahmung:* 2., 7., 12.

*Rückfälligkeit:* 3., 8., 13.

*Leidensdruck:* 4., 9., 14.

*Negative Konsequenzen:* 5., 10., 15., 16., 17., 18., 19.

Appendix 3. Assumption tests of multivariate analysis

Before primary analyses, the data were investigated for the assumptions of multivariate analyses based on detailed guidelines (Field, 2009). More specifically, univariate normality (i.e., the inspection of skewness and kurtosis values) was not achieved based on pre-established guidelines (Muthén & Kaplan, 1985). Skewness values ranged between -0.62 and 4.02 in Sample 1, between -0.09 and 4.19 in Sample 2, between -0.01 and 2.84 in Sample 3, and between -.029 and 4.95 in Sample 4 (see Appendix 4). Kurtosis values ranged between -0.89 and 17.33 in Sample 1, between -1.30 and 18.13 in Sample 2, between -1.26 and 7.35 in Sample 3, and between -1.04 and 55.38 in Sample 4 (see Appendix 4). Mardia’s two-sided tests for multivariate normality were significant (all *p*s < .001) in each sample, supporting the violation of multivariate normality (Wang & Wang, 2012). Nevertheless, the Durbin-Watson test suggested the independence of the residuals (i.e., all values were close to two). Also, we tested linearity and homoscedasticity (i.e., we examined scatterplots, histograms, and P-P plots of the residuals). In sum, besides normality, all other assumptions were met.

Appendix 4. Table 2. Initial item set of the Compulsive Sexual Behavior Disorder Scale (CSBD-19) with normality indices and corrected item-total correlations on each sample

|  | **Sample 1 (N = 7,995)** | | | | | **Sample 2 (N = 473)** | | | | | | **Sample 3 (N = 477)** | | | | | | **Sample 4 (N = 380)** | | | | |
| --- | --- | --- | --- | --- | --- | --- | --- | --- | --- | --- | --- | --- | --- | --- | --- | --- | --- | --- | --- | --- | --- | --- |
| **Factors and items** | Sk | SE | K | SE | CITC | Sk | SE | K | SE | CITC | Sk | | SE | K | SE | CITC | Sk | | SE | K | SE | CITC |
| **I. Control** |  |  |  |  |  |  |  |  |  |  |  | |  |  |  |  |  | |  |  |  |  |
| 1. I engaged in sexual activities that I knew I would later regret. (HBI4) | 1.06 | .03 | -0.31 | .05 | .39 | 1.81 | .11 | 2.04 | .22 | .43 | 1.35 | | .11 | 0.62 | .22 | .49 | 1.30 | | .13 | 0.66 | .25 | .22 |
| 1. **Even though my sexual behavior was irresponsible or reckless, I found it difﬁcult to stop. (HBI11)** | 1.26 | .03 | 0.26 | .05 | .63 | 1.84 | .11 | 2.29 | .22 | .68 | 1.97 | | .11 | 3.02 | .22 | .77 | 2.09 | | .13 | 3.33 | .25 | .62 |
| 1. **I could not control my sexual cravings and desires.** | 1.12 | .03 | 0.12 | .05 | .64 | 1.76 | .11 | 2.17 | .22 | .69 | 1.86 | | .11 | 2.78 | .22 | .74 | 1.82 | | .13 | 2.84 | .25 | .66 |
| 1. My sex life was out of control. | 2.06 | .03 | 3.75 | .05 | .61 | 2.30 | .11 | 4.76 | .22 | .72 | 2.76 | | .11 | 7.35 | .22 | .64 | 4.16 | | .13 | 18.40 | .25 | .58 |
| 1. When I craved sex, I had to act on it. | 1.12 | .03 | 0.25 | .05 | .48 | 1.28 | .11 | 0.53 | .22 | .54 | 0.97 | | .11 | -0.13 | .22 | .52 | 0.97 | | .13 | -0.30 | .25 | .48 |
| 1. **My sexual desires controlled me.** | 1.31 | .03 | 0.74 | .05 | .61 | 1.82 | .11 | 2.47 | .22 | .74 | 1.85 | | .11 | 2.49 | .22 | .71 | 1.96 | | .13 | 3.31 | .25 | .60 |
| **II. Salience** |  |  |  |  |  |  |  |  |  |  |  | |  |  |  |  |  | |  |  |  |  |
| 1. **Sex has been the most important thing in my life.** | 0.70 | .03 | -0.33 | .05 | .53 | 0.82 | .11 | -0.23 | .22 | .46 | 1.06 | | .11 | 0.41 | .22 | .44 | 0.95 | | .13 | 0.50 | .25 | .48 |
| 1. **I would rather have had sex than to have done anything else.** | 1.10 | .03 | 0.04 | .05 | .55 | 1.68 | .11 | 2.01 | .22 | .60 | 1.29 | | .11 | 0.92 | .22 | .64 | 0.30 | | .13 | -1.14 | .25 | .52 |
| 1. **When I could have sex, everything else became irrelevant.** | 0.64 | .03 | -0.89 | .05 | .60 | 0.95 | .11 | -0.39 | .22 | .61 | 1.49 | | .11 | 1.15 | .22 | .63 | 1.26 | | .13 | 0.56 | .25 | .57 |
| 1. My sexual desires and sexual activities were the only things mattered. | 2.32 | .03 | 5.10 | .05 | .55 | 2.86 | .11 | 8.40 | .22 | .56 | 2.37 | | .11 | 4.99 | .22 | .55 | 3.77 | | .13 | 14.67 | .25 | .42 |
| 1. I often thought how good it would be to engage in sexual activities. | -0.62 | .03 | -0.38 | .05 | .50 | -0.09 | .11 | -1.30 | .22 | .54 | -0.01 | | .11 | -1.26 | .22 | .42 | -0.29 | | .13 | -1.04 | .25 | .53 |
| 1. I continually planned when to engage in sexual activities. | 0.85 | .03 | -0.42 | .05 | .64 | 1.37 | .11 | 0.87 | .22 | .71 | 1.12 | | .11 | 0.08 | .22 | .63 | 1.39 | | .13 | 0.87 | .25 | .36 |
| **III. Relapse** |  |  |  |  |  |  |  |  |  |  |  | |  |  |  |  |  | |  |  |  |  |
| 1. Attempts to change my sexual behavior failed. (HBI7mod) | 1.17 | .03 | 0.27 | .05 | .43 | 1.32 | .11 | 0.66 | .22 | .56 | 1.48 | | .11 | 1.09 | .22 | .59 | 1.02 | | .13 | -0.09 | .25 | .29 |
| 1. **I was able to resist my sexual urges for only a little while before I surrendered to them.** | 1.14 | .03 | 0.10 | .05 | .67 | 1.62 | .11 | 1.76 | .22 | .74 | 0.95 | | .11 | -0.32 | .22 | .63 | 0.79 | | .13 | -0.66 | .25 | .57 |
| 1. I unsuccessfully tried to resist my sexual urges. | 1.17 | .03 | 0.21 | .05 | .71 | 1.59 | .11 | 1.58 | .22 | .74 | 1.45 | | .11 | 0.97 | .22 | .72 | 2.08 | | .13 | 3.66 | .25 | .63 |
| 1. Even if I tried to regulate my sexual desires, I did not succeed. | 1.59 | .03 | 1.53 | .05 | .74 | 1.95 | .11 | 2.94 | .22 | .78 | 2.04 | | .11 | 3.43 | .22 | .73 | 2.42 | | .13 | 5.59 | .25 | .68 |
| 1. **Trying to reduce the amount of sex I had almost never worked.** | 1.65 | .03 | 1.78 | .05 | .65 | 2.11 | .11 | 4.00 | .22 | .77 | 1.94 | | .11 | 2.95 | .22 | .71 | 2.54 | | .13 | 5.94 | .25 | .67 |
| 1. **I was not successful in reducing the amount of sex I had.** | 2.04 | .03 | 3.46 | .05 | .69 | 2.59 | .11 | 6.56 | .22 | .77 | 1.69 | | .11 | 1.87 | .22 | .72 | 2.67 | | .13 | 6.51 | .25 | .67 |
| **IV. Dissatisfaction** |  |  |  |  |  |  |  |  |  |  |  | |  |  |  |  |  | |  |  |  |  |
| 1. Sex has not been satisfying for me anymore. | 2.15 | .03 | 4.21 | .05 | .46 | 1.87 | .11 | 2.82 | .22 | .44 | 1.17 | | .11 | 0.18 | .22 | .67 | 1.31 | | .13 | 0.86 | .25 | .60 |
| 1. I engaged in sex, although I did not find it satisfying anymore. | 1.31 | .03 | 0.64 | .05 | .67 | 1.22 | .11 | 0.48 | .22 | .72 | 1.50 | | .11 | 1.07 | .22 | .76 | 1.11 | | .13 | -0.01 | .25 | .77 |
| 1. I had sex even when it did not bring me real satisfaction. | 1.37 | .03 | 0.77 | .05 | .73 | 1.65 | .11 | 1.70 | .22 | .73 | 1.08 | | .11 | -0.07 | .22 | .76 | 1.22 | | .13 | 0.28 | .25 | .81 |
| 1. **I had sex even when I did not enjoy it anymore.** | 2.05 | .03 | 3.48 | .05 | .74 | 1.89 | .11 | 2.61 | .22 | .79 | 1.50 | | .11 | 1.03 | .22 | .81 | 1.62 | | .13 | 1.50 | .25 | .77 |
| 1. **Although sex was not as satisfying for me as before, I engaged in it.** | 1.35 | .03 | 0.73 | .05 | .77 | 1.30 | .11 | 0.59 | .22 | .82 | 1.15 | | .11 | 0.01 | .22 | .79 | 1.37 | | .13 | 0.66 | .25 | .77 |
| 1. **Although my sex life was not as satisfying as it had been before, I had sex.** | 1.53 | .03 | 1.29 | .05 | .75 | 1.56 | .11 | 1.36 | .22 | .78 | 0.94 | | .11 | -0.38 | .22 | .70 | 1.41 | | .13 | 0.69 | .25 | .77 |
| **V. Negative consequences** |  |  |  |  |  |  |  |  |  |  |  | |  |  |  |  |  | |  |  |  |  |
| **V/1. General negative consequences** |  |  |  |  |  |  |  |  |  |  |  | |  |  |  |  |  | |  |  |  |  |
| 1. My sexual urges and impulses negatively affected me. | 1.71 | .03 | 2.16 | .05 | .58 | 1.86 | .11 | 2.97 | .22 | .62 | 1.73 | | .11 | 2.41 | .22 | .68 | 1.59 | | .13 | 1.79 | .25 | .57 |
| 1. My sexual behaviors had negative effects on my life. | 1.97 | .03 | 3.19 | .05 | .73 | 2.01 | .11 | 3.35 | .22 | .75 | 1.99 | | .11 | 2.94 | .22 | .76 | 2.09 | | .13 | 3.69 | .25 | .63 |
| 1. My sexual urges and behaviors had negative consequences. | 1.94 | .03 | 2.95 | .05 | .68 | 2.18 | .11 | 4.07 | .22 | .74 | 2.12 | | .11 | 3.56 | .22 | .74 | 2.16 | | .13 | 3.89 | .25 | .65 |
| 1. **My sexual urges and impulses changed me in a negative way.** | 2.50 | .03 | 5.90 | .05 | .68 | 2.58 | .11 | 6.38 | .22 | .74 | 2.17 | | .11 | 3.83 | .22 | .75 | 3.11 | | .13 | 1.28 | .25 | .70 |
| 1. The positives of sex in my life were far outweighed by the negatives. | 2.44 | .03 | 5.54 | .05 | .52 | 2.01 | .11 | 3.18 | .22 | .61 | 0.85 | | .11 | -0.84 | .22 | .20 | 1.55 | | .13 | 0.97 | .25 | .23 |
| 1. My sexual behavior compromised important areas of my life. | 2.97 | .03 | 8.84 | .05 | .62 | 3.17 | .11 | 9.97 | .22 | .66 | 1.97 | | .11 | 2.86 | .22 | .72 | 3.05 | | .13 | 9.50 | .25 | .63 |
| **V/2. General neglect** |  |  |  |  |  |  |  |  |  |  |  | |  |  |  |  |  | |  |  |  |  |
| 1. **I did not accomplish important tasks because of my sexual behavior.** | 1.59 | .03 | 1.78 | .05 | .69 | 1.97 | .11 | 3.54 | .22 | .73 | 2.09 | | .11 | 3.67 | .22 | .69 | 1.89 | | .13 | 2.96 | .25 | .65 |
| 1. I neglected tasks because of my sexual behavior. | 1.63 | .03 | 1.76 | .05 | .73 | 2.18 | .11 | 4.13 | .22 | .73 | 1.82 | | .11 | 2.36 | .22 | .79 | 1.81 | | .13 | 2.59 | .25 | .69 |
| 1. I did not accomplish requirements that were expected from me because of my sexual behavior. | 2.14 | .03 | 4.07 | .05 | .71 | 2.40 | .11 | 5.47 | .22 | .68 | 2.36 | | .11 | 5.08 | .22 | .79 | 3.01 | | .13 | 9.52 | .25 | .61 |
| 1. I failed to keep important commitments because of my sexual activities (HBCS2mod). | 2.75 | .03 | 7.53 | .05 | .75 | 3.08 | .11 | 1.18 | .22 | .73 | 2.52 | | .11 | 5.52 | .22 | .80 | 4.13 | | .13 | 19.51 | .25 | .71 |
| 1. Nothing else in my life mattered except for sex. | 1.62 | .03 | 2.06 | .05 | .51 | 2.16 | .11 | 4.22 | .22 | .61 | 2.43 | | .11 | 5.45 | .22 | .66 | 4.95 | | .13 | 3.12 | .25 | .52 |
| 1. Important goals were sacrificed because of my sexual activities. (HBCS7) | 2.59 | .03 | 6.71 | .05 | .64 | 3.22 | .11 | 11.24 | .22 | .64 | 2.30 | | .11 | 4.48 | .22 | .82 | 4.44 | | .13 | 22.21 | .25 | .56 |
| **V/3. Domain specific negative consequences** |  |  |  |  |  |  |  |  |  |  |  | |  |  |  |  |  | |  |  |  |  |
| V/3.1. Work and school |  |  |  |  |  |  |  |  |  |  |  | |  |  |  |  |  | |  |  |  |  |
| 1. **My sexual activities interfered with my work and/or education. (HBCS12mod)** | 1.93 | .03 | 3.04 | .05 | .75 | 2.23 | .11 | 4.27 | .22 | .76 | 2.33 | | .11 | 4.76 | .22 | .73 | 1.75 | | .13 | 2.12 | .25 | .72 |
| 1. My sexual behavior prevented me from bringing out the best in me occupationally and/or educationally. | 2.12 | .03 | 3.91 | .05 | .76 | 2.50 | .11 | 6.19 | .22 | .73 | 2.18 | | .11 | 3.90 | .22 | .75 | 2.38 | | .13 | 5.72 | .25 | .70 |
| 1. I dealt less with my occupation and/or education than I should have to because of my sexual behavior. | 1.92 | .03 | 2.78 | .05 | .78 | 2.26 | .11 | 4.58 | .22 | .77 | 2.27 | | .11 | 4.43 | .22 | .78 | 2.12 | | .13 | 3.51 | .25 | .79 |
| V/3.2. Relationships |  |  |  |  |  |  |  |  |  |  |  | |  |  |  |  |  | |  |  |  |  |
| 1. I emotionally hurt someone I cared about because of my sexual activities that I could not control. | 1.68 | .03 | 1.68 | .05 | .55 | 1.77 | .11 | 2.08 | .22 | .62 | 2.37 | | .11 | 4.91 | .22 | .70 | 2.26 | | .13 | 4.45 | .25 | .49 |
| 1. Due to my sexual behaviors, I was confronted by the people I loved. | 2.12 | .03 | 3.78 | .05 | .59 | 2.19 | .11 | 4.11 | .22 | .69 | 2.55 | | .11 | 5.92 | .22 | .61 | 1.79 | | .13 | 2.18 | .25 | .47 |
| 1. **My sexual behaviors had negative impact on my relationships with others.** | 2.33 | .03 | 5.07 | .05 | .53 | 2.45 | .11 | 5.64 | .22 | .63 | 2.28 | | .11 | 4.62 | .22 | .64 | 2.13 | | .13 | 3.83 | .25 | .58 |
| V/3.3. Personal feelings |  |  |  |  |  |  |  |  |  |  |  | |  |  |  |  |  | |  |  |  |  |
| 1. My self-respect, self-esteem, and/or self-confidence, was negatively impacted by my sexual activities. (HBCS19mod) | 1.27 | .03 | 0.55 | .05 | .49 | 1.52 | .11 | 1.38 | .22 | .62 | 1.46 | | .11 | 0.97 | .22 | .63 | 1.27 | | .13 | 0.46 | .25 | .40 |
| 1. I often felt anxious because I could not control my sexual behaviors. | 1.62 | .03 | 1.66 | .05 | .40 | 1.78 | .11 | 2.32 | .22 | .58 | 2.24 | | .11 | 4.44 | .22 | .67 | 3.76 | | .13 | 15.33 | .25 | .49 |
| 1. **I have been upset because of my sexual behaviors.** | 2.28 | .03 | 4.62 | .05 | .54 | 2.50 | .11 | 5.71 | .22 | .67 | 1.69 | | .11 | 1.74 | .22 | .75 | 1.81 | | .13 | 2.39 | .25 | .50 |
| V/3.4. Health and personal care |  |  |  |  |  |  |  |  |  |  |  | |  |  |  |  |  | |  |  |  |  |
| 1. I did not sleep enough because of my sexual behavior. | 1.19 | .03 | 0.19 | .05 | .23 | 1.61 | .11 | 1.71 | .22 | .34 | 1.65 | | .11 | 1.59 | .22 | .47 | 1.15 | | .13 | 0.14 | .25 | .17 |
| 1. I got a sexually transmitted disease or infection because of my sexual activities. (HBCS4) | 3.27 | .03 | 1.14 | .05 | .18 | 4.19 | .11 | 18.13 | .22 | .23 | 2.84 | | .11 | 7.11 | .22 | .54 | 4.06 | | .13 | 16.07 | .25 | .19 |
| 1. **My sexual activities interfered with my ability to experience healthy sex. (HBCS11)** | 1.94 | .03 | 2.80 | .05 | .24 | 2.09 | .11 | 3.39 | .22 | .42 | 2.14 | | .11 | 3.56 | .22 | .58 | 2.28 | | .13 | 4.48 | .25 | .18 |
| V/3.5. Other important areas of functioning |  |  |  |  |  |  |  |  |  |  |  | |  |  |  |  |  | |  |  |  |  |
| 1. **I often found myself in an embarrassing situation because of my sexual behavior.** | 1.96 | .03 | 3.30 | .05 | .50 | 2.32 | .11 | 5.26 | .22 | .72 | 2.02 | | .11 | 3.24 | .22 | .72 | 2.11 | | .13 | 4.39 | .25 | .25 |
| 1. I felt ashamed because of my sexual behavior. | 1.91 | .03 | 2.81 | .05 | .46 | 2.58 | .11 | 6.39 | .22 | .64 | 1.70 | | .11 | 1.70 | .22 | .64 | 1.95 | | .13 | 2.78 | .25 | .24 |
| 1. I experienced unwanted financial difficulties because of my sexual activities. (HBCS8mod) | 4.02 | .03 | 17.33 | .05 | .26 | 3.47 | .11 | 13.30 | .22 | .47 | 2.79 | | .11 | 7.25 | .22 | .62 | 7.32 | | .13 | 55.38 | .25 | .16 |

*Note*. CITC = Corrected Item-Total Correlation; Sk = skewness; K = kurtosis; SE = standard error. Items from the Hypersexual Behavior Inventory (HBI) and from the Hypersexual Behavior Consequences Scale (HBCS) were considered as potential items of the CSBD-19. Items from these scales are indicated with the abbreviation of the scale and the number of the item from the original scale (e.g., HBCS11). If these items were modified (e.g., present tense changed to past tense), the coding of the item includes “mod” at the end of the label (e.g., HBCS8mod). Bold letters indicate the final items.

Appendix 5. Table 3. Inter-factor correlations between the total score and the factors of the Compulsive Sexual Behavior Disorder Scale (CSBD-19) on each sample

| **Sample 1 (N = 7,995)** | 1. | 2. | 3. | 4. | 5. |
| --- | --- | --- | --- | --- | --- |
| 1. CSBD-19 total score | — |  |  |  |  |
| 2. CSBD-19 control | .86* | — |  |  |  |
| 3. CSBD-19 salience | .75* | .58* | — |  |  |
| 4. CSBD-19 relapse | .84* | .68* | .57* | — |  |
| 5. CSBD-19 dissatisfaction | .65* | .39* | .26* | .41* | — |
| 6. CSBD-19 negative consequences | .80* | .68* | .44* | .60* | .50* |
| **Sample 2 (N = 473)** | 1. | 2. | 3. | 4. | 5. |
| 1. CSBD-19 total score | — |  |  |  |  |
| 2. CSBD-19 control | .88* | — |  |  |  |
| 3. CSBD-19 salience | .74* | .57* | — |  |  |
| 4. CSBD-19 relapse | .87* | .75* | .63* | — |  |
| 5. CSBD-19 dissatisfaction | .75* | .52* | .35* | .52* | — |
| 6. CSBD-19 negative consequences | .85* | .77* | .48* | .70* | .60* |
| **Sample 3 (N = 477)** | 1. | 2. | 3. | 4. | 5. |
| 1. CSBD-19 total score | — |  |  |  |  |
| 2. CSBD-19 control | .89* | — |  |  |  |
| 3. CSBD-19 salience | .71* | .58* | — |  |  |
| 4. CSBD-19 relapse | .88* | .79* | .62* | — |  |
| 5. CSBD-19 dissatisfaction | .66* | .43* | .19* | .41* | — |
| 6. CSBD-19 negative consequences | .90* | .83* | .55* | .76* | .53* |
| **Sample 4 (N = 380)** | 1. | 2. | 3. | 4. | 5. |
| 1. CSBD-19 total score | — |  |  |  |  |
| 2. CSBD-19 control | .86* | — |  |  |  |
| 3. CSBD-19 salience | .67* | .49* | — |  |  |
| 4. CSBD-19 relapse | .83* | .76* | .49* | — |  |
| 5. CSBD-19 dissatisfaction | .58* | .32* | .07 | .28* | — |
| 6. CSBD-19 negative consequences | .84* | .74* | .45* | .65* | .42* |

*Note.* CSBD-19 = Compulsive Sexual Behavior Disorder Scale. * *p* < .01.

Appendix 6. Table 4. Fit indices for the latent profile analyses on the Compulsive Sexual Behavior Disorder Scale (CSBD-19) (N = 9,325)

| Classes | AIC | CAIC | BIC | SSABIC | Entropy | L-M-R Test | *p* |
| --- | --- | --- | --- | --- | --- | --- | --- |
| 1 | 132331.02 | 132412.42 | 132402.42 | 132370.64 | - | - | - |
| 2 | 113764.41 | 113894.66 | 113878.66 | 113827.81 | .93 | 18245.91 | < .001 |
| 3 | 108823.28 | 109002.37 | 108980.37 | 108910.46 | .91 | 4864.43 | < .001 |
| 4 | 106766.76 | 106994.69 | 106966.69 | 106877.71 | .92 | 2031.48 | < .001 |
| 5 | 105125.10 | 105401.87 | 105367.87 | 105259.83 | .91 | 1624.05 | .006 |
| **6** | **103418.29** | **103743.91** | **103703.91** | **103576.80** | **.91** | **1688.03** | **< .001** |
| 7 | 102316.44 | 102690.90 | 102644.90 | 102498.72 | .91 | 1093.91 | .267 |
| 8 | 101465.75 | 101889.06 | 101837.06 | 101671.81 | .91 | 847.235 | .057 |
| 9 | 100473.70 | 100945.85 | 100887.85 | 100703.54 | .91 | 953.23 | .305 |
| 10 | 99389.66 | 99910.65 | 99846.65 | 99643.27 | .92 | 878.45 | .140 |

*Note.* Classes = number of latent classes; AIC = Akaike Information Criterion; CAIC = corrected Akaike Information Criterion; BIC = Bayesian Information Criterion; SSABIC = Sample-Size Adjusted Bayesian Information Criterion; L-M-R test = The Lo-Mendell-Rubin Adjusted Likelihood Ratio Test; *p* = *p* value associated with the L-M-R Test. Bold letters indicate that the six-class solution was selected as the final model.

Appendix 7. Figure 1. Latent classes based on the factors of the Compulsive Sexual Behavior Disorder Scale (CSBD-19) on the merged sample (N = 9325). *Note.* Scores on the factors of the CSBD-19 were standardized (M = 0; SD = 1) to make scores comparable on the factors of the CSBD-19.

**
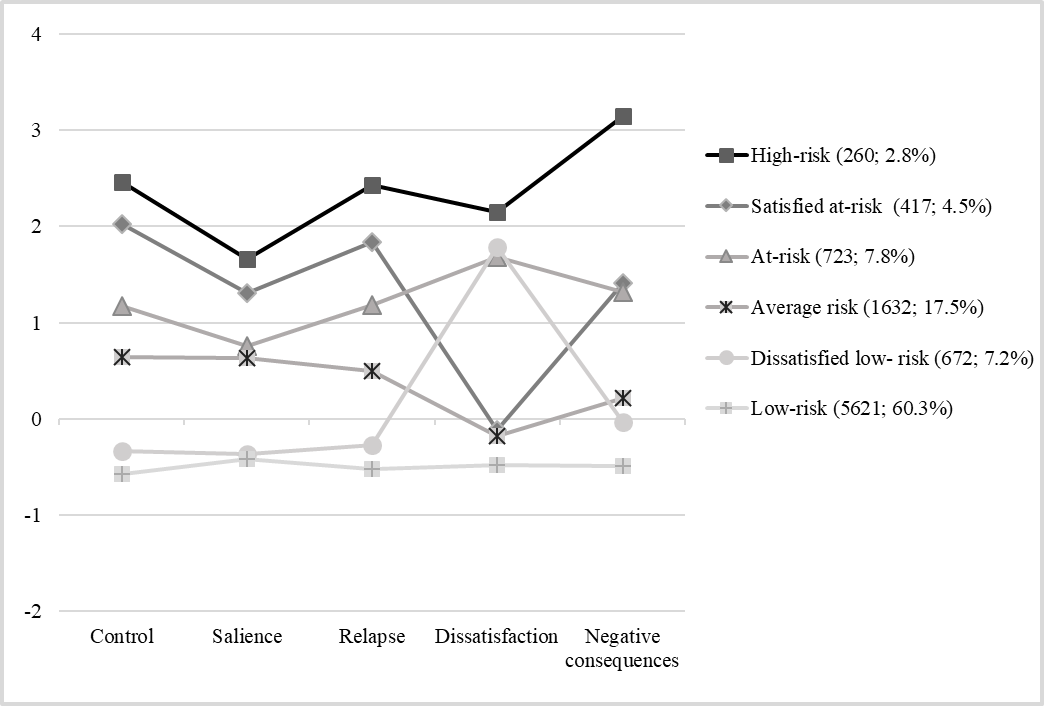
**

Appendix 8. Table 5. Calculation of cut-off thresholds for the Compulsive Sexual Behavior Disorder Scale (CSBD-19) (N = 9325)

| cut-off score | true positive | true negative | false positive | false negative | sensitivity (%) | specificity (%) | PPV (%) | NPV (%) | accuracy (%) |
| --- | --- | --- | --- | --- | --- | --- | --- | --- | --- |
| 46 | 260 | 8748 | 317 | 0 | 100% | 96.5% | 45.1% | 100% | 96.6% |
| 47 | 259 | 8824 | 241 | 1 | 99.6% | 97.3% | 51.8% | 100% | 97.4% |
| 48 | 259 | 8888 | 177 | 1 | 99.6% | 98.0% | 59.4% | 100% | 98.1% |
| 49 | 258 | 8946 | 119 | 2 | 99.2% | 98.7% | 68.4% | 100% | 98.7% |
| **50** | **256** | **8986** | **79** | **4** | **98.5%** | **99.1%** | **76.4%** | **100%** | **99.1%** |
| 51 | 244 | 9023 | 42 | 16 | 93.8% | 99.5% | 84.1% | 99.8% | 99.3% |
| 52 | 227 | 9041 | 24 | 33 | 87.3% | 99.7% | 90.4% | 99.6% | 99.4% |
| 53 | 213 | 9050 | 15 | 47 | 81.9% | 99.8% | 93.4% | 99.5% | 99.3% |
| 54 | 184 | 9054 | 11 | 76 | 70.8% | 99.9% | 94.4% | 99.2% | 99.1% |
| 55 | 151 | 9057 | 8 | 109 | 58.1% | 99.9% | 95.0% | 98.8% | 98.8% |

*Note.* The bolded row indicates the suggested cut-off threshold. Possible scores on CSBD-19 range from 19 to 76.

**References**

Field, A. (2009). *Discovering statistics using SPSS* (Third). doi: 10.1234/12345678

Kraus, S. W., Krueger, R. B., Briken, P., First, M. B., Stein, D. J., Kaplan, M. S., … Reed, G. M. (2018). Compulsive sexual behaviour disorder in the ICD-11. *World Psychiatry*, *17*(1), 109–110. doi: 10.1002/wps.20499

Muthén, B., & Kaplan, D. (1985). A comparison of some methodologies for the factor analysis of non-normal Likert variables. *British Journal of Mathematical and Statistical Psychology*, *38*(2), 171–189. doi: 10.1111/j.2044-8317.1985.tb00832.x

Wang, J., & Wang, X. (2012). *Structural equation modeling*. Chichester, UK: Wiley.
